# Dichloro­phosphinic bis­(2-chloro­eth­yl)amide

**DOI:** 10.1107/S1600536812049586

**Published:** 2012-12-08

**Authors:** Erqun Song, Yang Song

**Affiliations:** a Key Laboratory of Luminescence and Real-Time Analysis, Ministry of Education, College of Pharmaceutical Sciences, Southwest University, Chong Qing 400716, People’s Republic of China

## Abstract

In the title compound, C_4_H_8_Cl_4_NOP, the two chloro­ethyl groups are not related by crystallographic symmetry. The difference in the conformation of the two groups is shown by their N—C—C—Cl torsion angles of 64.57 (15) and 175.62 (10)°.

## Related literature

The title compound is a precursor used in the synthesis of the anti­tumor drug cyclo­phosphamide and its analogues. For information on organo­phospho­rus heterocyclic compounds, see: Surendra Babu *et al.* (2009 ▶); Srinivasulu *et al.* (2008 ▶); Krishna *et al.* (2006 ▶). For the crystal structures of cyclo­phosphamide analogues, see: Camerman & Camerman (1973 ▶); Jones *et al.* (1996 ▶); Himes *et al.* (1982 ▶); Camerman *et al.* (1983 ▶); Perales & García-Blanco (1977*a* ▶,*b* ▶); Gałdecki & Głowka (1981 ▶); Boyd *et al.* (1980 ▶); Shih *et al.* (1986 ▶). For the pharma­cological activity of cyclo­phosphamide analogues, see: Lin *et al.* (1980 ▶); Borch & Canute (1991 ▶).
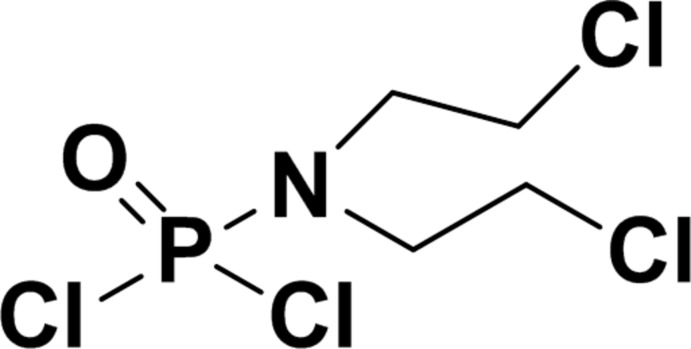


## Experimental

### 

#### Crystal data

C_4_H_8_Cl_4_NOP
*M* *_r_* = 258.88
Monoclinic, 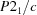

*a* = 9.0723 (15) Å
*b* = 8.4810 (14) Å
*c* = 13.135 (2) Å
β = 101.221 (2)°
*V* = 991.4 (3) Å^3^
*Z* = 4
Mo *K*α radiation
μ = 1.30 mm^−1^
*T* = 298 K
0.16 × 0.12 × 0.10 mm

#### Data collection

Bruker APEXII CCD diffractometer
Absorption correction: multi-scan (*SADABS*; Bruker, 2009 ▶) *T*_min_ = 0.819, *T*_max_ = 0.881
9480 measured reflections
3255 independent reflections
2725 reflections with *I* > 2σ(*I*)
*R*_int_ = 0.020

#### Refinement

*R*[*F* ^2^ > 2σ(*F* ^2^)] = 0.029
*wR*(*F* ^2^) = 0.090
*S* = 1.05
3255 reflections
101 parameters
H-atom parameters constrained
Δρ_max_ = 0.57 e Å^−3^
Δρ_min_ = −0.46 e Å^−3^

### 

Data collection: *APEX2* (Bruker, 2009 ▶); cell refinement: *SAINT* (Bruker, 2009 ▶); data reduction: *SAINT*; program(s) used to solve structure: *SHELXS97* (Sheldrick, 2008 ▶); program(s) used to refine structure: *SHELXL97* (Sheldrick, 2008 ▶); molecular graphics: *SHELXTL* (Sheldrick, 2008 ▶); software used to prepare material for publication: *SHELXTL* and local procedures.

## Supplementary Material

Crystal structure: contains datablock(s) I, global. DOI: 10.1107/S1600536812049586/fy2076sup1.cif

Supplementary material file. DOI: 10.1107/S1600536812049586/fy2076Isup2.cdx

Structure factors: contains datablock(s) I. DOI: 10.1107/S1600536812049586/fy2076Isup3.hkl

Supplementary material file. DOI: 10.1107/S1600536812049586/fy2076Isup4.cml

Additional supplementary materials:  crystallographic information; 3D view; checkCIF report

## Figures and Tables

**Fig. 1.**
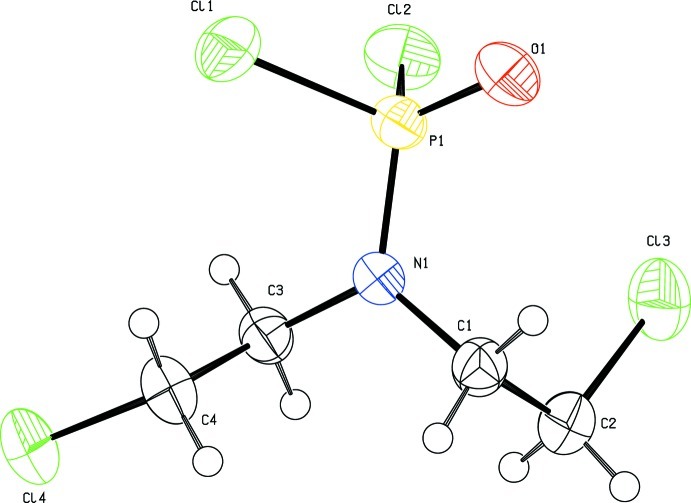
View of the title compound showing the atom-labelling scheme. Displacement ellipsoids are drawn at the 30% probability level.

**Fig. 2.**
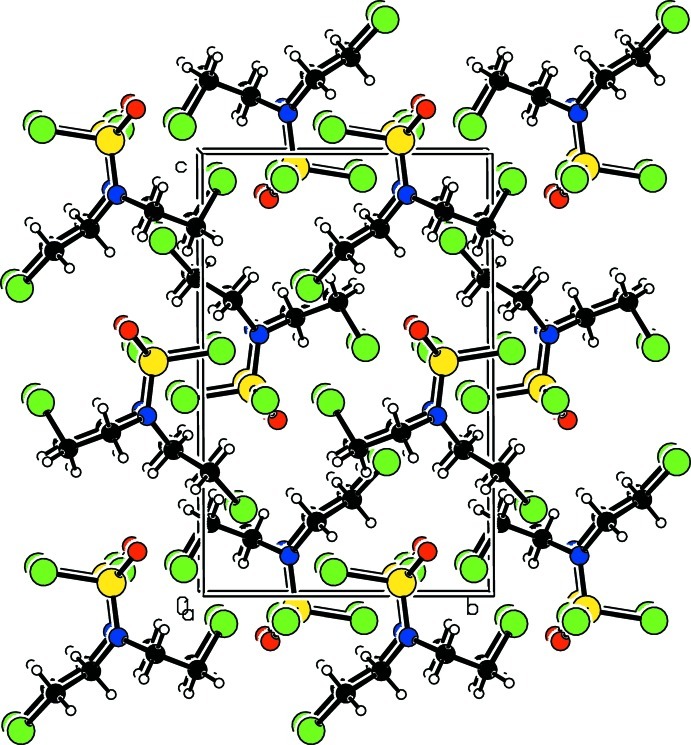
Crystal packing of title compound, viewed approximately down the a axis, illustrating the stacking of the molecules along the a axis.

## supplementary crystallographic information

### Comment

Cyclophosphamide, a nitrogen mustard alkylating agent, is widely used as an anti-cancer agent. It is converted in the liver to its active form, which is dependent on cytochrome P450 metabolism for its therapeutic effectiveness. During the process of metabolism, a toxic byproduct, acrolein is generated and induces hemorrhagic cystitis. Many cyclophosphamide analogues were developed to reduce this side effect and to find more potent anti-cancer drugs. The title compound is used as an important precursor for the synthesis of cyclophosphamide and its analogues.

In the title molecule (I) (Fig. 1), all bond lengths and angles are within normal ranges and correspond to those observed in related compounds. Atoms C3, N1, P1 and O1 are nearly coplanar, with a dihedral angel of 175.23 (10)° between the C3—N1—P1 and N1—P1—O1 planes. Angles for C3—N—P, P—N—C1 and C1—N—C3 are 120.57 (9)°, 121.02 (9)° and 118.04 (11)°, respectively. It is interesting to notice that two 2-chloroethyls are not symmetry-related, the torsion angle of N1—C1—C2—Cl3is 64.57 (15)°, but the torsion angle of N1—C3—C4—Cl4 is 175.62 (10)°. Although the current compound is conformationally flexible, twist conformation isomers form on closing the phospho-heterocycle, as, for example, in cyclophosphamide (Borch *et al.*, 1991).

### Experimental

Bis(2-chloroethyl)amine hydrochloride (20.0 g, 0.112 mol) was added dropwise into a 250 ml round bottom bottle containing POCl_3_ (52 ml, 0.6 mol). Then the mixture was refluxed for 20 h at 110 °C. The disappearance of solid bis(2-chloroethyl)amine hydrochloride indicated completion of the reaction. To remove excess POCl_3_, reduced vacuum was used. The crude were dissolved into ethyl acetate and the precipitate was filtered off. The filtrate was concentrated in vacuo and the resulting residue was recrystallized with acetone and hexane (*v*/*v* = 1:5), giving white crystals (18.0 g) in a yield of 61%. Single crystals for X-ray diffraction were grown at room temperature by slow evaporation from the solution of the title compound in ethanol.

### Refinement

The H-atoms bonded to C-atoms were positioned geometrically and refined using a riding model, with C—H = 0.93 Å, and with *U*_iso_(H) = 1.2 *U*_eq_(C).

### Crystal data

**Table:**

C_4_H_8_Cl_4_NOP	*F*(000) = 520
*M**_r_* = 258.88	*D*_x_ = 1.735 Mg m^−^^3^
Monoclinic, *P*2_1_/*c*	Mo *K*α radiation, λ = 0.71073 Å
*a* = 9.0723 (15) Å	Cell parameters from 4728 reflections
*b* = 8.4810 (14) Å	θ = 2.3–31.1°
*c* = 13.135 (2) Å	µ = 1.30 mm^−^^1^
β = 101.221 (2)°	*T* = 298 K
*V* = 991.4 (3) Å^3^	Block, colourless
*Z* = 4	0.16 × 0.12 × 0.10 mm

### Data collection

**Table:**

Bruker APEXII CCD diffractometer	3255 independent reflections
Radiation source: fine-focus sealed tube	2725 reflections with *I* > 2σ(*I*)
Graphite monochromator	*R*_int_ = 0.020
φ and ω scans	θ_max_ = 32.2°, θ_min_ = 2.3°
Absorption correction: multi-scan (*SADABS*; Bruker, 2009)	*h* = −13→13
*T*_min_ = 0.819, *T*_max_ = 0.881	*k* = −12→12
9480 measured reflections	*l* = −19→13

### Refinement

**Table:**

Refinement on *F*^2^	Secondary atom site location: difference Fourier map
Least-squares matrix: full	Hydrogen site location: inferred from neighbouring sites
*R*[*F*^2^ > 2σ(*F*^2^)] = 0.029	H-atom parameters constrained
*wR*(*F*^2^) = 0.090	*w* = 1/[σ^2^(*F*_o_^2^) + (0.0482*P*)^2^ + 0.1876*P*] where *P* = (*F*_o_^2^ + 2*F*_c_^2^)/3
*S* = 1.05	(Δ/σ)_max_ = 0.002
3255 reflections	Δρ_max_ = 0.57 e Å^−^^3^
101 parameters	Δρ_min_ = −0.46 e Å^−^^3^
0 restraints	Extinction correction: *SHELXL97* (Sheldrick, 2008), Fc^*^=kFc[1+0.001xFc^2^λ^3^/sin(2θ)]^-1/4^
Primary atom site location: structure-invariant direct methods	Extinction coefficient: 0.0064 (13)

### Special details

**Table:**

Geometry. All e.s.d.'s (except the e.s.d. in the dihedral angle between two l.s. planes) are estimated using the full covariance matrix. The cell e.s.d.'s are taken into account individually in the estimation of e.s.d.'s in distances, angles and torsion angles; correlations between e.s.d.'s in cell parameters are only used when they are defined by crystal symmetry. An approximate (isotropic) treatment of cell e.s.d.'s is used for estimating e.s.d.'s involving l.s. planes.
Refinement. Refinement of *F*^2^ against ALL reflections. The weighted *R*-factor *wR* and goodness of fit *S* are based on *F*^2^, conventional *R*-factors *R* are based on *F*, with *F* set to zero for negative *F*^2^. The threshold expression of *F*^2^ > σ(*F*^2^) is used only for calculating *R*-factors(gt) *etc*. and is not relevant to the choice of reflections for refinement. *R*-factors based on *F*^2^ are statistically about twice as large as those based on *F*, and *R*- factors based on ALL data will be even larger.

### Fractional atomic coordinates and isotropic or equivalent isotropic displacement parameters (Å^2^)

**Table:**

	*x*	*y*	*z*	*U*_iso_*/*U*_eq_	
C1	0.38633 (16)	0.82538 (16)	0.87493 (12)	0.0381 (3)	
H1A	0.4301	0.7845	0.8186	0.046*	
H1B	0.4682	0.8544	0.9310	0.046*	
C2	0.2970 (2)	0.97152 (18)	0.83776 (13)	0.0467 (3)	
H2A	0.2125	0.9432	0.7834	0.056*	
H2B	0.3599	1.0447	0.8087	0.056*	
C3	0.19521 (15)	0.60676 (16)	0.83359 (10)	0.0353 (3)	
H3A	0.1429	0.6769	0.7802	0.042*	
H3B	0.1208	0.5556	0.8662	0.042*	
C4	0.27918 (18)	0.48303 (19)	0.78420 (13)	0.0448 (3)	
H4A	0.3374	0.4169	0.8376	0.054*	
H4B	0.3479	0.5337	0.7463	0.054*	
Cl1	0.31341 (5)	0.44237 (4)	1.05878 (3)	0.05244 (12)	
Cl3	0.22968 (5)	1.06456 (5)	0.94101 (4)	0.05789 (13)	
Cl2	0.08981 (4)	0.71564 (5)	1.05773 (3)	0.05103 (12)	
Cl4	0.14781 (6)	0.36563 (5)	0.69800 (4)	0.06145 (14)	
N1	0.29789 (12)	0.69934 (13)	0.91169 (9)	0.0328 (2)	
O1	0.41231 (13)	0.76345 (14)	1.10623 (8)	0.0474 (3)	
P1	0.29887 (4)	0.67627 (4)	1.03420 (3)	0.03356 (10)	

### Atomic displacement parameters (Å^2^)

**Table:**

	*U*^11^	*U*^22^	*U*^33^	*U*^12^	*U*^13^	*U*^23^
C1	0.0403 (6)	0.0353 (6)	0.0409 (7)	−0.0035 (5)	0.0131 (5)	−0.0024 (5)
C2	0.0600 (9)	0.0347 (6)	0.0451 (8)	−0.0025 (6)	0.0094 (7)	0.0020 (6)
C3	0.0376 (6)	0.0343 (6)	0.0330 (6)	0.0004 (5)	0.0042 (5)	−0.0035 (5)
C4	0.0481 (8)	0.0409 (7)	0.0444 (8)	−0.0001 (6)	0.0067 (6)	−0.0131 (6)
Cl1	0.0676 (3)	0.03754 (19)	0.0523 (2)	0.00726 (15)	0.01214 (19)	0.01047 (15)
Cl3	0.0608 (3)	0.0414 (2)	0.0733 (3)	0.00801 (16)	0.0174 (2)	−0.01022 (18)
Cl2	0.04327 (19)	0.0641 (3)	0.0497 (2)	0.00731 (16)	0.01884 (16)	−0.00236 (17)
Cl4	0.0777 (3)	0.0514 (2)	0.0521 (3)	−0.0121 (2)	0.0049 (2)	−0.01980 (19)
N1	0.0376 (5)	0.0315 (5)	0.0292 (5)	−0.0022 (4)	0.0062 (4)	−0.0026 (4)
O1	0.0479 (6)	0.0540 (6)	0.0369 (5)	−0.0032 (5)	−0.0006 (4)	−0.0072 (5)
P1	0.03567 (17)	0.03484 (17)	0.02971 (16)	0.00247 (12)	0.00520 (12)	−0.00150 (12)

### Geometric parameters (Å, º)

**Table:**

C1—N1	1.4732 (18)	C3—H3A	0.9700
C1—C2	1.509 (2)	C3—H3B	0.9700
C1—H1A	0.9700	C4—Cl4	1.7800 (16)
C1—H1B	0.9700	C4—H4A	0.9700
C2—Cl3	1.7767 (17)	C4—H4B	0.9700
C2—H2A	0.9700	Cl1—P1	2.0100 (6)
C2—H2B	0.9700	Cl2—P1	2.0081 (6)
C3—N1	1.4713 (16)	N1—P1	1.6195 (12)
C3—C4	1.515 (2)	O1—P1	1.4567 (11)
			
N1—C1—C2	114.16 (12)	H3A—C3—H3B	108.0
N1—C1—H1A	108.7	C3—C4—Cl4	109.25 (11)
C2—C1—H1A	108.7	C3—C4—H4A	109.8
N1—C1—H1B	108.7	Cl4—C4—H4A	109.8
C2—C1—H1B	108.7	C3—C4—H4B	109.8
H1A—C1—H1B	107.6	Cl4—C4—H4B	109.8
C1—C2—Cl3	111.13 (11)	H4A—C4—H4B	108.3
C1—C2—H2A	109.4	C3—N1—C1	118.04 (11)
Cl3—C2—H2A	109.4	C3—N1—P1	120.57 (9)
C1—C2—H2B	109.4	C1—N1—P1	121.02 (9)
Cl3—C2—H2B	109.4	O1—P1—N1	116.78 (7)
H2A—C2—H2B	108.0	O1—P1—Cl2	112.63 (5)
N1—C3—C4	111.43 (11)	N1—P1—Cl2	108.07 (5)
N1—C3—H3A	109.3	O1—P1—Cl1	112.37 (5)
C4—C3—H3A	109.3	N1—P1—Cl1	105.45 (4)
N1—C3—H3B	109.3	Cl2—P1—Cl1	100.01 (2)
C4—C3—H3B	109.3		
			
N1—C1—C2—Cl3	64.57 (15)	C3—N1—P1—O1	175.23 (10)
N1—C3—C4—Cl4	175.62 (10)	C1—N1—P1—O1	−11.87 (13)
C4—C3—N1—C1	78.71 (15)	C3—N1—P1—Cl2	−56.60 (10)
C4—C3—N1—P1	−108.18 (13)	C1—N1—P1—Cl2	116.30 (10)
C2—C1—N1—C3	75.10 (16)	C3—N1—P1—Cl1	49.65 (10)
C2—C1—N1—P1	−97.97 (14)	C1—N1—P1—Cl1	−137.45 (10)
